# Hydroxychloroquine and COVID-19: can we learn from the use of rituximab in systemic lupus erythematosus?

**DOI:** 10.11604/pamj.2021.38.372.29087

**Published:** 2021-04-15

**Authors:** Fernando Kemta Lekpa, Bertrand Hugo Mbatchou Ngahane, Sylvain Raoul Simeni Njonnou, Hermine Fouda, Marie Patrice Halle, Yacouba Mapoure Njankouo, Anastase Dzudie, Simeon Pierre Choukem, Henry Namme Luma

**Affiliations:** 1 Internal Medicine Department, Douala General Hospital, Douala, Cameroon,; 2 Department of Internal Medicine and Specialties, Faculty of Medicine and Pharmaceutical Sciences, University of Dschang, Dschang, Cameroon,; 3 The University of Dschang Taskforce for the Elimination of COVID-19 (UNITED#COVID-19), Dschang, Cameroon,; 4 Health and Human Development (2HD) Research Network, Douala, Cameroon

**Keywords:** Hydroxychloroquine, rituximab, COVID-19, randomized controlled trials

## Abstract

Rituximab (RTX), a chimeric monoclonal anti-CD20 antibody has become part of the standard therapy for patients with CD20-expressing B-cell lymphoma and rheumatoid arthritis. After encouraging results with open studies in systemic lupus erythematosus (SLE), RTX has not shown its effectiveness in randomized controlled trials. However, its efficacy has been validated in renal, hematological, and neuropsychiatric disorders. Understanding the history of RTX in SLE would be instructive in the hydroxychloroquine (HCQ) saga in COVID-19. Three steps would be necessary and sufficient before definitively closing the debate: 1) determine the effective and safe dose of HCQ, as well as the minimum duration of treatment in COVID-19; 2) define the profile of patients in whom HCQ would be more likely to be effective (especially in asymptomatic patients and/or at the onset of the first signs of the disease) and 3) conduct one or more multicentre RCT to evaluate the efficacy and safety of HCQ in COVID-19 in SSA.

## Commentary

Sir, Rituximab (RTX), a chimeric monoclonal anti-CD20 antibody, has become part of the standard therapy for patients with CD20-expressing B-cell lymphoma and rheumatoid arthritis. The first data from observational studies had given satisfactory results in patients with systemic lupus erythematosus (SLE) refractory to conventional treatments [1]. These results have not always been confirmed by randomized controlled trials (RCT). However, positive results have been described for some locations in this non-specific systemic organ auto-immune disease. Thus, RTX is currently used off-label, particularly in patients with severe renal, hematological and/or neuropsychiatric disease refractory to other immunosuppressive therapies or in patients with contraindications to these drugs [2]. More so, RTX is included in the 2019 updates of the European League Against Rheumatism (EULAR) recommendations for the management of SLE [3].

Although hydroxychloroquine (HCQ) is recommended for all patients with SLE [3], this drug has found a new usage with the COVID-19 disease. This COVID-19 made HCQ the most publicized drug of 2020 [4]. After the positive results from observational studies on HCQ in COVID-19 [5], wide use of this drug has been observed worldwide [4]. Subsequently, RCT, systematic reviews and meta-analysis with contradictory results were also published. The Cochrane review declared HCQ persona non grata in COVID-19, thus signing game over by showing that HCQ has little or no effect on the risk of death and probably no effect on the progression to mechanical ventilation for people infected with COVID-19 [6].

HCQ with or without azithromycin has been widely used as a treatment protocol for COVID-19 by many countries in SSA, in the absence of controlled trials and with different dosage (Table 1) [7, 8]. The same is true for some herbal medicines which have been widely used in the absence of evidence of their effectiveness [9]. The limited access to therapeutic resources in SSA [10] leads us to ask ourselves once again the following question: apart from the young age of Africans, is the low mortality in SSA linked to the wide use of herbal medicines and dietary therapy or the result of the effectiveness of HCQ in COVID-19 in this population? To find out, it would be imperative not to consider the recommendation of the Cochrane review to no longer conduct further trials on HCQ for the treatment of COVID-19, but to conduct clinical trials on available drugs and herbal medicines accessible in SSA at a cost that the community and African countries can afford.

**Table 1:** different dosages of hydroxychloroquine in COVID-19 used at the Douala General Hospital, Douala, Cameroon*

Date	Hydroxychloroquine, 200 mg
From March 2020 to May 2020	1 tablet three times a day for 10 days
From June 2020 to August 2020	1 tablet three times a day for 5 to 7 days
From September 2020 to February 2020	1 tablet twice a day for 5 days
From March 2020 to date	1 tablet three times a day for 5 to 7 days

* Between April 2020 and March 2020, the doses of hydroxychloroquine (associated with azithromycin) were determined by the endowment offered by the Ministry of Health of Cameroon.

Understanding the history of RTX in SLE would be instructive in the HCQ saga in COVID-19. In the absence of data on the populations of SSA from the “Solidarity” clinical trial, three steps would be necessary and sufficient before definitively closing the debate: 1) determine the effective and safe dose of HCQ, as well as the minimum duration of treatment in COVID-19; 2) define the profile of patients in whom HCQ would be more likely to be effective (especially in asymptomatic patients and/or at the onset of the first signs of the disease); and 3) conduct one or more multicentre RCT to evaluate the efficacy and safety of HCQ in COVID-19 in SSA.
